# Life’s Late Digital Revolution and Why It Matters for the Study of the Origins of Life

**DOI:** 10.3390/life7030034

**Published:** 2017-08-25

**Authors:** David A. Baum, Niles Lehman

**Affiliations:** 1 Department of Botany, University of Wisconsin, 430 Lincoln Drive, Madison, WI 53706, USA; 2 Department of Chemistry, Portland State University, P.O. Box 751, Portland, OR 97207, USA

**Keywords:** origins of life, information, digital, genetic inheritance

## Abstract

The information contained in life exists in two forms, analog and digital. Analog information is manifest mainly in the differing concentrations of chemicals that get passed from generation to generation and can vary from cell to cell. Digital information is encoded in linear polymers such as DNA and RNA, whose side chains come in discrete chemical forms. Here, we argue that the analog form of information preceded the digital. Acceptance of this dichotomy, and this progression, can help direct future studies on how life originated and initially complexified on the primordial Earth, as well as expected trajectories for other, independent origins of complex life.

## 1. Introduction

Approximately four billion years ago, life emerged in some unknown chemical milieu on the Earth and, as Darwin put it in the last sentence of the *Origin of Species*, “from so simple a beginning endless forms most beautiful and most wonderful have been, and are being, evolved.” This amazing fact demands explanation. How was life “originally breathed into a few forms or into one,” and how did life come to be sufficiently evolvable to be able to give rise to such endless forms? We suggest that the original chemical self-organization, the breath of life if you will, should be seen as a distinct event from a later transition that conferred high evolvability, namely, the emergence of digital inheritance.

Though not the binary 0/1 of the computer chip, life is now digitally encoded in the quaternary A/C/G/T of the chromosome [1,2]. This fact is so central to our conception of modern biology that the decoding of life’s digital genetic code constitutes the main plot line in any chronicle of the history of biology: from Mendel’s particulate inheritance, to Watson and Crick’s double helix, to Nirenberg’s genetic code. Therefore, it is not surprising that the origins-of-life field has been rather obsessed with the chicken-and-egg problem: How could a digital computer as complex as the genetic system have arisen ab initio? Which came first, the software (genes) or the hardware (metabolism)?

We argue in this essay that life began in analog mode, which is to say as a system in which a parent cell passes on its characteristics to its daughter cells by giving them a set of chemicals whose concentrations are similar to that of the parent cell. Analog systems can be simpler than digital ones because there is no clear distinction between software and hardware. This insight offers the prospect of making great progress on the origin of life problem by dividing it up into two easier questions: How did an analog system capable of adaptive evolution first arise? And, how and why did a digital system, with its much greater evolutionary potential, get layered on top? Both questions are much more easily answered once we recognize that they are distinct.

## 2. Digital and Analog Life

To begin, consider the benefits that the digital system confers on living organisms. As first argued by Schrödinger [3], though not using the digital-analog terminology, organisms as complex as those we see today would not be able to faithfully pass on a phenotype to their offspring if they were purely analog. The problem is that cells are small and contain very many different, essential biochemicals, such that some chemicals are represented by only a few molecules per cell. During division, such rare chemical species would experience high fluctuations in concentration, meaning that a daughter cell would be very unlikely to have all important molecules at suitable concentrations. The fact that living cells achieve faithful replication despite this led Schrödinger to deduce that some aspect of inheritance must exploit the quantum properties of covalent bonds to reduce sampling variance. He predicted, perspicaciously as we now know, that inheritance must be based on an “aperiodic crystal.”

Schrödinger’s claim—that any entity as small as a bacterial cell and with as many distinct components as the simplest bacterium could not persist through multiple divisions without digital encoding—is unassailable. However, his assertion also has limits. It does not rule out analog inheritance co-existing with digital encoding in living cells, and it does not rule out some kinds of purely analog systems being viable, for example ones that have a higher volume or fewer distinct chemical moieties. Both of these limitations need to be considered.

For all the centrality of genetic inheritance, it is clear that life is only partially digital [2]. Developmental and physiological functions during a cell’s lifetime are largely analog, depending on the relative and absolute concentrations of different cellular chemicals. Less obvious, but still certain, is that generation-to-generation inheritance has a major analog component. Daughter cells are formed with allocations of membranes and key biomolecules whose local and global concentrations need to fall within certain bounds for the daughter cells to function. Morowitz has argued that life is present in the 3D spatial arrangement of atoms in living cells [4]. This is why it is so hard to form a cell de novo even from a complete genome of DNA [5]. Regardless of whether there might be an engineered solution to this limitation of synthetic biology, it is a reasonable assumption that cellular life has always had an analog component. But must life have also always had digital information encoding? Does our notion of “life” allow for purely analog inheritance?

While every definition of life has some imperfections, most would agree that the ability to evolve adaptively in such a way that complexity can increase is a necessity for something to be considered alive. Sample published definitions include: “… an organism, to be called living, must be capable of both replication and mutation; such an organism will evolve into higher forms” [6]; “Life is a self-sustained chemical system capable of undergoing Darwinian evolution” [7]; and, “… an autonomous system with open-ended evolutionary capacities” [8]. Our task is to determine whether any form of life can be analog and still conform to the intent of these definitions.

Imagine a membrane-bound cell that lacked a digital genetic system but could nonetheless grow and divide. Such a cell would contain a set of chemical species that are reflexively autocatalytic, meaning that they cooperate with one another to acquire resources and energy from the environment and turn them into more of the same chemicals [9,10]. After such a parent cell divides, each daughter cell would be expected to contain chemicals at a concentration close to the parent cell. Provided that all chemical species needed for growth and division were represented by at least some thousands of molecules per cell, which is quite possible if the cell is not too small and if the number of distinct chemicals is not too high, each daughter cell would have a composition close enough to the parent cell to expect them to inherit the ability to grow and divide. Through such compositional inheritance [9], daughter cells inherit their parents’ features. However, although daughters will be similar to parents, they will rarely be identical, because chance events during cell division will yield deviations in the concentrations of some chemicals [11]. These differences can be thought of as the analog equivalent to mutations. While they might often make the daughter cell less able than its parent to grow and divide, once in a while the mutant might be better at doing so, in which case its descendant cells would tend to be overrepresented. Consequently, natural selection might be expected to cause progressive changes to a population of dividing analog cells [9].

It turns out that the assertion that a purely analog cell can evolve adaptively is oversimplified. Vasas et al. [12] showed that the rate at which compositional genomes change is too high for progressive evolution. Fortunately for our argument, the claim that composition alone is too mutagenic for natural selection to be effective leaves out an important feature of living metabolism—homeostasis. An autocatalytic system that is able to exploit a replenishing supply of food and energy to grow, and thereby achieve dynamic-kinetic stability [13], represents a metastable attractor state surrounded by a zone of attraction. As long as chemical concentrations remain in the zone of attraction, the system is homeostatic: if one chemical in the autocatalytic set falls to a low level, it tends to be replenished, and if one becomes overabundant, it tends to diminish. Homeostatic mechanisms of this sort mean that small fluctuations in concentration are corrected, allowing for more faithful inheritance from generation to generation than is possible by composition alone [14]. Such a mechanism will reduce the effective analog mutation rate, but mutations will still occur when changes in chemical concentration are sufficient to bounce the system as a whole to a new metastable equilibrium, which might have higher or lower fitness than the prior state. This shows that analog systems can achieve the noise reduction needed for adaptive evolution, but instead of depending on covalent bonds to generate discrete states, analog living systems use the homeostatic tendencies of metastable dynamic attractor states. Such a perspective suggests that the original function of the digital genetic system that life uses might have been homeostatic control over biochemical concentrations, as can be seen in modern systems. Thanks to genetic encoding, a cell can completely lack certain RNA or protein molecules, yet its daughter cells can generate them as needed. Or, at a more basic level, an RNA species forms a mini autocatalytic cycle with inherent homeostatic capabilities: in conditions permissive for template-guided synthesis, the RNA and its complementary sequence can each bounce back from very low concentrations.

To summarize, all known life has a major analog component, suggesting that analog systems are at least as old as the last universal common ancestor. At the same time we have shown that living systems can, in principle, function in a purely analog mode and that analog systems are potentially much simpler than digital ones. As a result, we have good grounds to explore the idea that life began in the analog state and only later went digital. Before asking why the digital revolution happened, let us first clarify constraints on the chemical basis of digital encoding systems.

## 3. The Role of Bond Strength in Life

Before life, there were no individuated organisms, and thus nascent life was necessarily a collection of spatially localized chemical reactions. These chemicals must have embodied some form of collective reproduction: an ability to increase in local concentration and expand into more space (i.e., grow). Furthermore, some chemical systems must have been superior at reproducing than other forms, implying some adaptive potential. However, only some chemicals involved in life had any potential for digital encoding.

Today, we tend to think of life as being dominated by linear polymers of information-bearing subunits. Polypeptides and polynucleotides come to mind immediately, although there are also possible precursor forms of these and many other chemical classes that share their essential features: a structural backbone and variable side chains. The backbone is structurally invariant or repetitive and does not contain information, while the side-chains imbue these molecules with the potential to encode digital information.

We posit that this duality between backbone and side-chains in informational polymers exists due to a synergy between the use of covalent and non-covalent bonds. Covalent bonds, being more permanent in an aqueous solution, allow for the persistence of genotypes. Thus, it is the sequences of side groups that tend to keep a living entity at a metastable equilibrium. However, this homeostasis has limits: genotypes exist long enough for natural selection to operate on them, but breaking and reforming covalent bonds also occurs and is the basis of genetic mutations. Non-covalent bonds between the side groups of different polymers, principally hydrogen bonds but also hydrophobic interactions, are very transient. These short-lived bonds are, however, central in allowing digitalization because they permit the transfer of information from one polymer to another. For example, in today’s nucleic acids the H-bond donor and acceptor patterns on the edges of nucleobases, when tuned by the pH of the surrounding solvent, act in a highly predicable manner to allow for high fidelity information transfer from one polymer to another [15]. For this to work, it is critical that the non-covalent bonds used to transfer information between side-chains are much weaker than the covalent bonds of the backbone. If this were not the case, the separation of the polymers after information transfer would be problematic. Think of the challenge of removing masking tape when the glue is as strong as the integrity of the tape itself. Note that this would be true regardless of the mechanism of reproduction, be it template-directed replication or some other form of copying such as ligation or recombination [16].

While modern RNA has an excellent balance between backbone stability and H-bond precision, there are many other potential ways to use covalent and non-covalent bonds to allow digital information transfer. Contemporary polypeptides do not display such precise H-bonding patterns because they have not been selected to serve as information bearers, but in principle they could be used for information transfer, as shown experimentally by Severin et al. [17]. Furthermore, there are good reasons to think that the set of H-bonding moieties that contemporary nucleic acids utilize is an evolved trait [18,19]. Of direct relevance, Krishnamurthy [19] has argued that the evolution of RNA as an information polymer required the pre-existence of heterogenous nucleic acid chemistry involving mixtures of multiple backbone monomers and nucleobases. Only through selection on polymer function for something other than genetic encoding could homogenous RNA, with a ribose backbone and four canonical bases capable of Watson-Crick hydrogen-bonding, become stabilized [19]. This implies that the origin of RNA itself occurred through a selective process that occurred without H-bond-mediated information transfer. It was only once template-mediated RNA-copying arose as a major determinant of fitness that digital inheritance at the molecular level could begin to take hold.

## 4. The Path to the Digital Cell

The existence of biopolymers suited for digitally encoding information in their side-chains is a necessary but not a sufficient condition for digital life as we know it. Consider a cell composed of many alternative RNA molecules. While each molecule digitally encodes information on its own properties, the properties of the cell as a whole—its phenotype—still depend on the numbers of copies of each RNA species that it contained. Thus, the inheritance of traits from a parental cell to its daughters would still be analog, depending on the concentrations of the RNA species. Such a cell would be subject to the Schrödinger constraint: the number of distinct RNA variants that it could pass on would be limited, because with too many variants some RNA species would necessarily become too rare to be reliably inherited. How could life have progressed to the modern mode of inheritance in which a single chromosome digitally encodes the RNA and protein repertoire, and hence phenotype, of an entire cell?

Theoretical work has explained how a fragmented genome composed of slowly-copied, non-functional, complementary RNA sequences would arise due to conflicts between RNA and cell fitness [20]. Additional models provide hints as to how the slowest-replicating, essential genomic sequence, perhaps composed of many covalently-linked genome fragments, might become the master regulator of cell division and eventually a chromosome [21]. Once cell phenotype came to be controlled chromosomally, life would have achieved the level of digitality seen today—not complete, but great enough for efficient adaptive evolution driven by digital mutations.

What about translation and the genetic code? Clearly, only with ribosomes or some other translation system could a nucleic acid genome control the repertoire of both RNA and protein species. However, this does not mean that translation is required for digital inheritance. Indeed, we suggest that the origin of ribosomes and the genetic code, fascinating as they are, might best be seen as contingent innovations that greatly expanded the adaptive potential of cellular life, rather than a necessary feature of digital life in general.

## 5. Analog “Life” Preceded Digital Life 

Adaptive evolution requires both heritability (offspring being very similar to their parents) and variation (occasional heritable changes in phenotype). In a digital system, mutations involve changes in the encoded information. In an analog system, mutations entail jumps from one metastable state to another, where a metastable equilibrium is a set of concentrations that are sustained at a constant value (or show deterministic fluctuations) by dynamical homeostasis. In multi-stable autocatalytic chemical networks with alternative metastable equilibria, as illustrated by the Belousov-Zhabotinsky reaction and other Brusselators, an analog mutation therefore corresponds to a change in concentration of one or several chemicals that is so great that the system leaves the current metastable state and finds another one. Analog systems can also change through the assimilation of new chemical components, which may initially arise from rare side reactions but then become amplified autocatalytically. While mechanistically different than a substitution or indel in a nucleic acid molecule, analog mutations have the essential properties needed for them to serve as the fuel for adaptive evolution: after they occur, they will tend to be passed on to subsequent generations and will be favored in that process if the mutant form enhances survival or reproduction. This indicates that fully analog systems can be adaptively evolvable. This allows that the earliest life could have lacked digital inheritance, yet have been poised to acquire digital encoding systems that conferred a fitness advantage.

Given the clear capacity for autocatalysis and multi-stability in diverse chemical mixtures, it is easy to imagine evolvable analog systems arising spontaneously in geological settings with reliable fluxes of food (spontaneously formed chemical building blocks) and energy [22]. However, while evolvable analog systems seem well-poised to bootstrap themselves into existence, the simplest digital encoding system known, template-directed replication of RNA molecules, is too complicated to have arisen without being situated in a system that was already evolving adaptively [16,22]. Thus, it seems almost inescapable that life began analog and only later acquired a digital aspect.

Recognizing that there were two sequential events, first the origin of an analog chemical system capable of adaptive evolution and then a digital revolution, the origin of life problem becomes much more tractable. Each step can be modeled and studied empirically, independent of the other. Theoretical analyses can be used to assess the conditions under which evolvable autocatalytic chemical systems would be expected to arise spontaneously. Prior work suggests that complex chemical mixtures have a high probability of containing autocatalytic cycles [23,24,25,26] and that such structures can emerge without specialized catalysts such as protein enzymes or ribozymes [27]. Furthermore, whereas the examples given earlier involved chemical systems enclosed in a membrane, theory suggests that adaptive evolution could act on autocatalytic systems associated with mineral surfaces such that areas occupying fitter (faster growing, more stable) metastable states could invade neighboring areas, resulting in fitter systems over time. Combined with selection for dispersal ability, there is also a relatively direct path from surface-associated life to compartmentalized life [22]. Importantly, by removing the need to incorporate either genetic encoding or compartmentalization at the onset of life, new empirical approaches suggest themselves. For instance, one could generate environments in the lab conducive to the emergence of surface-associated autocatalytic systems and then look for evidence of adaptive evolution [28].

When it comes to explaining the origin of digital encoding, the challenge is much reduced when one allows that it arose in fully analog organisms, probably already compartmentalized into cells, that were able to evolve adaptively. For example, it seems feasible to model (and even study in vitro) gradual increases in the accuracy of template-guided RNA synthesis to better understand the threshold at which digital inheritance can become a target of natural selection. In fact, constructing models of RNA evolution that do not rely on template-directed polymerization [16] may help us rationalize why Nature settled on the four canonical nucleobases. A particular goal of such work should be to evaluate whether specific polypeptide catalysts are needed to achieve the required fidelity of RNA copying, because this would dictate whether RNA control of peptide sequences (i.e., translation) evolved in the analog or digital phase, either of which is a priori possible. Additionally, it seems feasible to explore the autocatalytic feedback between polynucleotides and nucleotide-generating metabolism (e.g., the Kreb’s cycle and glycolysis) to better understand why RNA rather than other polymers emerged as critical information and energy carriers. This could extend prior work [18,19] to assess whether the centrality of RNA is historically contingent or due to RNA having the perfect balance of hydrogen-bonding potential and covalent trans-phosphorylation potential to emerge as a digital information bearer in a cytoplasmic environment. Lastly, the separation of analog and digital stages of life could help resolve the question of whether RNA preceded proteins, or whether they co-evolved [29].

## 6. Conclusions

The origin of life itself has typically been viewed as requiring at least one major transition of very low probability. Yet to explain the simultaneous origin of growing and dividing cellular compartments and a digital genetic encoding system would require two such events to occur concomitantly. Recognizing that life almost certainly went digital much after it was already evolving adaptively helps lessen the extreme improbability of its origin. Evolvable analog systems could self-organize with high probability and then permit the gradual acquisition of digital genetic encoding, first at the molecular and then at the cellular levels. We believe that separating the analog and digital steps represents a significant change of focus that can help scientists sharpen their understanding of origins of life and develop new, productive empirical research programs.

## References

[1] Emmeche C., Hoffmeyer J. (1991). From langauge to nature: The semiotic metaphor in biology. Semiotica.

[2] Walker S.I., Davies P.C.W. (2013). The algorithmic origins of life. J. R. Soc. Interface.

[3] Schrödinger E. (1944). What Is Life?.

[4] Skoultchi A.I., Morowitz H.J. (1964). Information storage and survival of biological systems at temperatures near absolute zero. Yale J. Biol. Med..

[5] Feitelson D.G., Treinin M. (2002). The blueprint for life?. Computer.

[6] Horowitz N.H., Miller S.L. (1962). Current Theories on the Origin of Life. Fortschritte der Chemie Organischer Naturstoffe/Progress in the Chemistry of Organic Natural Products/Progrès dans la Chimie des Substances Organiques Naturelles.

[7] Joyce G.F., Deamer D.W., Fleischaker G.R. (1994). Foreword. Origins of Life: The Central Concepts.

[8] Ruiz-Mirazo K., Peretó J., Moreno A. (2004). A universal definition of life: Autonomy and open-ended evolution. Orig. Life Evol. Biosph..

[9] Segrè D., Ben-Eli D., Lancet D. (2000). Compositional genomes: Prebiotic information transfer in mutually catalytic noncovalent assemblies. Proc. Natl. Acad. Sci. USA.

[10] Shenhav B., Segrè D., Lancet D. (2003). Mesobiotic emergence: Molecular and ensemble complexity in early evolution. Adv. Complex Syst..

[11] Szathmáry E., Demeter L. (1987). Group selection of early replicators and the origin of life. J. Theor. Biol..

[12] Vasas V., Szathmáry E., Santos M. (2010). Lack of evolvability in self-sustaining autocatalytic networks constraints metabolism-first scenarios for the origin of life. Proc. Natl. Acad. Sci. USA.

[13] Pross A. (2011). Toward a general theory of evolution: Extending Darwinian theory to inanimate matter. J. Syst. Chem..

[14] Markovitch O., Lancet D. (2012). Excess mutual catalysis is required for effective evolvability. Artif. Life.

[15] Krishnamurthy R. (2012). Role of p*K*_a_ of nucleobases in the origins of chemical evolution. Acc. Chem. Res..

[16] Lehman N. (2008). A recombination-based model for the origin and early evolution of genetic information. Chem. Biodivers..

[17] Severin K., Lee D.H., Martinez J.A., Ghadiri M.R. (1997). Peptide self-replication via template-directed ligation. Chem. Eur. J..

[18] Eschenmoser A. (1999). Chemical etiology of nucleic acid structure. Science.

[19] Krishnamurthy R. (2015). On the emergence of RNA. Isr. J. Chem..

[20] Takeuchi N., Hogeweg P., Kaneko K. (2017). The origin of a primordial genome through spontaneous symmetry breaking. Nat. Commun..

[21] Kamimura A., Kaneko K. (2010). Reproduction of a protocell by replication of a minority molecule in a catalytic reaction network. Phys. Rev. Lett..

[22] Baum D.A. (2015). Selection and the origin of cells. Bioscience.

[23] Mossel E., Steel M. (2005). Random biochemical networks: The probability of self-sustaining autocatalysis. J. Theor. Biol..

[24] Kauffman S.A. (1986). Autocatalytic sets of proteins. J. Theor. Biol..

[25] Kauffman S.A. (1971). Cellular homeostasis, epigenesis and replication in randomly aggregated macromolecular systems. J. Cybern..

[26] Hordijk W., Steel M., Kauffman S.A. (2012). The structure of autocatalytic sets: Evolvability, enablement, and emergence. Acta Biotheor..

[27] Virgo N., Ikegami T. (2013). Autocatalysis before enzymes: The emergence of prebiotic chain reactions. Adv. Artif. Life ECAL.

[28] Baum D.A., Vetsigian K. (2016). An experimental framework for generating evolvable chemical systems in the laboratory. Orig. Life Evol. Biosph..

[29] Kovacs N.A., Petrov A.S., Lanier K.A., Williams L.D. (2017). Frozen in time: The history of proteins. Mol. Biol. Evol..

